# Circulating CD5L is associated with cardiovascular events and all-cause mortality in individuals with chronic kidney disease

**DOI:** 10.18632/aging.203615

**Published:** 2021-10-10

**Authors:** Esmeralda Castelblanco, Maria R. Sarrias, Àngels Betriu, Berta Soldevila, Maria Barranco-Altirriba, Josep Franch-Nadal, Jose M. Valdivielso, Marcelino Bermudez-Lopez, Per-Henrik Groop, Elvira Fernández, Núria Alonso, Didac Mauricio

**Affiliations:** 1 Department of Endocrinology and Nutrition, Hospital de la Santa Creu i Sant Pau and Sant Pau Biomedical Research Institute (IIB Sant Pau), Barcelona 08041, Spain; 2 Department of Internal Medicine, Endocrinology, Metabolism and Lipid Research Division, Washington University School of Medicine, St Louis, MO 63110, USA; 3 Institute for Health Science Research Germans Trias i Pujol (IGTP) and Center for Biomedical Research on Liver and Digestive Diseases (CIBEREHD), Madrid 28029, Spain; 4 Vascular and Renal Translational Research Group, Biomedical Research Institute of Lleida, (IRBLleida), Lleida 25198, Spain; 5 Department of Endocrinology and Nutrition, Health Sciences Research Institute and University Hospital Germans Trias i Pujol, Badalona 08916, Spain; 6 B2SLab, Department of Systems Engineering, Automation and Industrial Informatics, Polytechnic University of Catalonia, Barcelona 08034, Spain; 7 DAP-Cat Group, Unitat de Suport a la Recerca Barcelona, Fundació Institut Universitari per a la recerca a l’Atenció Primària de Salut Jordi Gol i Gurina (IDIAPJGol), Barcelona 08006, Spain; 8 Department of Medicine, Barcelona Autonomous University (UAB), Bellaterra 08193, Spain; 9 Primary Health Care Center Raval Sud, Gerència d’Atenció Primaria, Institut Català de la Salut, Barcelona 08001, Spain; 10 Folkhälsan Institute of Genetics, Folkhälsan Research Center, Helsinki 00290, Finland; 11 Abdominal Center Nephrology, University of Helsinki and Helsinki University Central Hospital, Helsinki 00280, Finland; 12 Research Program for Clinical and Molecular Metabolism, Faculty of Medicine, University of Helsinki, Helsinki 00100, Finland; 13 Department of Diabetes, Central Clinical School, Monash University, Melbourne, Victoria 3004, Australia; 14 Faculty of Medicine, University of Vic – Central University of Catalonia (UVic/UCC), Vic 08500, Spain; 15 Center for Biomedical Research on Diabetes and Associated Metabolic Diseases (CIBERDEM), Barcelona 08907, Spain

**Keywords:** CD5L, sCD36, chronic kidney disease, cardiovascular events, mortality

## Abstract

This study assessed the association of CD5L and soluble CD36 (sCD36) with the risk of a cardiovascular event (CVE), including CV death and all-cause mortality in CKD. We evaluated the association of CD5L and sCD36 with a predefined composite CV endpoint (unstable angina, myocardial infarction, transient ischemic attack, cerebrovascular accident, congestive heart failure, arrhythmia, peripheral arterial disease [PAD] or amputation by PAD, aortic aneurysm, or death from CV causes) and all-cause mortality using Cox proportional hazards regression, adjusted for CV risk factors. The analysis included 1,516 participants free from pre-existing CV disease followed up for 4 years. The median age was 62 years, 38.8% were female, and 26.8% had diabetes. There were 98 (6.5%) CVEs and 72 (4.8%) deaths, of which 26 (36.1%) were of CV origin. Higher baseline CD5L concentration was associated with increased risk of CVE (HR, 95% CI, 1.17, 1.0–1.36), and all-cause mortality (1.22, 1.01–1.48) after adjusting for age, sex, diabetes, systolic blood pressure, dyslipidemia, waist circumference, smoking, and CKD stage. sCD36 showed no association with adverse CV outcomes or mortality. Our study showed for the first time that higher concentrations of CD5L are associated with future CVE and all-cause mortality in individuals with CKD.

## INTRODUCTION

Chronic kidney disease (CKD) is a well-known independent risk factor for premature cardiovascular disease (CVD) and death [1]. In individuals with CKD, cardiovascular (CV) events and mortality increase progressively with declining renal function and/or increasing albuminuria [1], with a 50% risk of CV mortality even before reaching end-stage renal disease [2].

CV events and mortality are only partially explained by the traditional risk factors of diabetes, dyslipidemia, hypertension, obesity, smoking, and gender. Furthermore, more recently described biomarkers have not improved the prediction of CV events in individuals with renal impairment [3–7]. As a result, current risk prediction algorithms may underestimate the CV risk in adults with CKD [8]. Therefore, the identification of novel and more efficient biomarkers for early CV risk prediction is essential to be able to implement optimal risk-reduction strategies to improve clinical outcomes.

CD5 molecule-like (CD5L), also known as apoptosis inhibitor of macrophages (AIM), is a 40-kDa secreted glycoprotein that belongs to the scavenger receptor cysteine-rich superfamily. It participates in a broad spectrum of biological mechanisms that control inflammatory responses involved in infections, atherosclerosis, and cancer [9, 10]. Additionally, CD5L modulates other aspects of macrophage biology, such as antimicrobial responses through Toll-like receptor activation [10, 11].

CD36 is an 88-kDa transmembrane glycoprotein expressed in a wide variety of cell types, with critical roles in macrophage metabolism, activation of transforming growth factor beta (TGF-β), and uptake of oxidized low-density lipoprotein (oxLDL). This receptor is associated with inflammation and stands at the crossroads of cardio- and cerebrovascular diseases [12]. Additionally, CD36 also plays a role in atherosclerosis progression [13, 14], and it is associated with traditional CV risk factors [15].

Both scavenger receptors, CD5L and CD36, are essential molecules related to inflammatory responses and atherosclerosis mediated by macrophages; while CD36 oxLDL endocytosis prompts foam cell formation, CD5L facilitates CD36-mediated oxLDL uptake [16]. Additionally, in adipose tissue, macrophage-derived CD5L taken up by adipocytes through CD36-mediated endocytosis, stimulates lipolysis. In turn, the lipolytic response stimulates adipocyte inflammation favoring the induction of metabolic disorders predisposing to severe CVD [17]. Based on these cellular functions, CD36 has been proposed as a biomarker of CVD [18], although the soluble form in plasma (sCD36) as a CVD predictive factor is a controversial issue. Indeed, some studies have reported that high levels of sCD36 represent a strong biomarker of CVD in individuals with diabetes and of CV mortality in people with CKD [19, 20], while others did not find any significant association with CV risk [21, 22]. Given these contradictory results and since the issue has not been fully explored in a large population of individuals with CKD, we hypothesized that high concentrations of CD5L and sCD36 could be useful biomarkers of an increased risk of CV events and mortality in individuals with CKD.

## RESULTS

The study included 1,516 CKD subjects followed for a median of 4.1 years (interquartile range [IQR], 3.7; 4.4). The characteristics of the study population are described in Table 1. Median age was 62 years (IQR, 51; 68), 38.9% (*n* = 590) of the participants were female, and 26.8% (*n* = 406) had diabetes. Almost all CKD subjects had hypertension (*n* = 1,392; 91.8%) and 69.5% (*n* = 1,053) had dyslipidemia. The etiology of CKD was diverse: in 21.2% of cases it was related to a vascular disease; in 15.7% to glomerular nephropathy; and in 14.6% to diabetic kidney disease (additional causes are in Supplementary Figure 1). Up to 240 (15.8%) participants received a kidney transplant during follow-up. For most variables, there were significant differences by gender except for body mass index, hypertension, dyslipidemia, and diastolic blood pressure (Supplementary Table 1). Regarding the potential biomarkers, there were no significant differences in the median CD5L concentration by gender (females: 2,230 ng/mL [IQR, 1,798; 2,888]; males: 2,295 ng/mL [IQR, 1,818; 2,875]). In contrast, sCD36 was significantly higher in females than in males (1.20 ng/mL [IQR, 0.05; 8.86] vs. 0.85 ng/mL [IQR, 0.05; 5.37]).

**Table 1:** Demographic and clinical characteristics of the study subjects.

	**CKD**
*N*	1516
Gender, female, *n* (%)	590 (38.92%)
Age, years, median [IQR]	62 [51; 68]
Diabetes mellitus, *n* (%)	406 (26.8%)
Body mass index, kg/m^2^, mean (SD)	28.7 (5.4)
Waist circumference, cm, mean (SD)	98.9 (12.9)
Active smoker, *n* (%)	292 (19.3%)
Hypertension, *n* (%)	1392 (91.8%)
Dyslipidemia, *n* (%)	1053 (69.5%)
Systolic blood pressure, mmHg, mean (SD)	142.8 (21.1)
Diastolic blood pressure, mmHg, mean (SD)	81.6 (11.3)
Creatinine, mg/dl, median [IQR]	2.02 [1.52; 2.89]
Albumin/creatinine ratio, mg/g, median [IQR]	103.5 [12.5; 455.5]
eGFR, mL/min/1,73 m^2^, median [IQR]	32.4 [21.9; 45.4]
CKD stage 3, *n* (%)	669 (44.1%)
CKD stage 4–5, *n* (%)	539 (35.6%)
Dialysis, *n* (%)	308 (20.3%)
Aspartate transaminase, U/L, median [IQR]	19 [16; 24]
Alanine transaminase, U/L, median [IQR]	19 [14; 27]
Total cholesterol, mg/dL, median [IQR]	177.2 [153; 205]
HDL cholesterol, mg/dL, median [IQR]	47 [39; 58]
LDL cholesterol, mg/dL, median [IQR]	101 [79; 122]
Triglycerides, mg/dL, median [IQR]	123 [92; 175]
Glucose, mg/dL, median [IQR]	98 [88; 113]
HbA1c, %, median [IQR]	5.9 [5.3; 6.7]
Hematocrit, %, median [IQR]	38.83 (5.09)
Hemoglobin, g/dL, mean (SD)	12.9 (1.73)
CD5L, ng‎/mL, median [IQR]	2276 [1812; 2876]
sCD36, ng/mL, median [IQR]	1.0 [0.05; 6.75]

Abbreviations: CKD: chronic kidney disease; eGFR: estimated glomerular filtration rate; HDL: high-density lipoprotein; IQR: interquartile range; LDL: low-density lipoprotein; SD: standard deviation.

The participants with diabetes were older (65.0 years [IQR, 58.0; 70.0] vs. 61.0 years [IQR, 50.0; 67.0]), had a higher median body mass index (30.0 kg/m^2^ [IQR, 26.5; 33.7] vs. 27.5 kg/m^2^ [IQR, 24.5; 30.9], waist circumference (103 cm [IQR, 94.0; 111] vs. 96.0 cm [IQR, 89.0; 105]), and systolic blood pressure (146 mmHg [IQR, 132; 162] vs. 139 mmHg [IQR, 127; 154]), and more often had hypertension (98.3% vs. 89.5%) and dyslipidemia (82.3% vs. 64.8%) (Supplementary Table 2). As for the biomarkers, the median CD5L concentrations were significantly higher among the participants with diabetes (2,421.2 ng/mL; IQR, 1,899; 3,105) than in those without diabetes (2,206.11ng/mL; IQR, 1,782; 2,802) (Supplementary Table 2). Conversely, the sCD36 concentrations were not significantly different between participants with or without diabetes (1.31 ng/mL [IQR, 0.05; 7.57] vs. 0.83 ng/mL [IQR, 0.05; 5.93]) (Supplementary Table 2).

### Incidence of cardiovascular events

A total of 98 (6.5%) CV events were registered during the follow-up, yielding a CV event rate of 1.55 per 1,000 person-years (specific causes are described in Supplementary Figure 2). Compared to participants without a CV event, those who did suffer a CV event were more frequently diabetic (43.9% vs. 25.6%), on dialysis therapy (29.6% vs. 19.7%), active smokers (29.6% vs. 18.5%), had a higher waist circumference (102 cm [IQR, 93;110] vs. 98.0 cm [IQR, 90; 107]), and lower HDL cholesterol concentrations (43.0 mg/dL [IQR, 33.5; 51.2] vs. 47.0 mg/dL [IQR, 39.0; 58.5]) (Supplementary Table 3). However, gender had no effect on the proportion of incident CV events (7.0% in males vs. 5.9% in females) or on the CV event rate, which was 1.69 per 1,000 person-years in men and 1.32 per 1000 person-years in females. Moreover, participants with diabetes had a higher rate of CV events (2.61 per 1,000 person-years) than those without diabetes (1.17 per 1,000 person-years). Finally, participants who had a CV event exhibited higher CD5L concentrations than those who did not (2,571 ng/mL [IQR, 1,910; 3,314] and 2,245 ng/mL [IQR, 1,806; 2,847], respectively; *p* = 0.004). Conversely, the sCD36 concentrations were not different between the groups (1.31 ng/mL [IQR, 0.05; 6.66] vs. 0.96 ng/mL [IQR, 0.05; 6.79]).

### All-cause mortality

A total of 72 deaths (6.5%) were registered during follow-up with a rate of 1.11 per 1,000 person-years. Among them, 26 (36.1%) were CV deaths with a rate of 0.4 per 1,000 person-years. CD5L and sCD36 concentrations were higher in those who died (*n* = 72), compared to the participants alive at the end of the follow-up period (CD5L: 2,409 [1,876; 3,546] vs. 2,255 [1,811; 2,857] ng/m, and CD36: 3.19 [0.17; 9.66] vs. 0.94 [0.05; 6.64] ng/mL). All-cause mortality was not significantly higher in males than in females (5.62% vs. 3.39%), with an all-cause mortality rate in men of 1.32 per 1,000 person-years and 0.79 per 1,000 person-years in women. In participants with diabetes, the proportion of deaths was higher than in those without diabetes (7.39% vs. 3.78%), the rate of all-cause mortality being 1.76 vs. 0.88 per 1,000 person-years, respectively.

### Factors associated with cardiovascular disease

Cox proportional hazards models revealed that CD5L was an independent predictor of CV events (hazard ratio [HR], 1.17; 95% confidence interval [CI], 1.0–1.36) after adjusting for CV risk factors such as age, sex, diabetes, waist circumference, smoking, systolic blood pressure, dyslipidemia, and CKD stage (Table 2). Other independent predictors of CV event risk were older age, diabetes, smoking habit, and dialysis therapy (Table 2). The results of the model for CD5L including estimated glomerular filtration rate (eGFR) are shown in Supplementary Table 4. In contrast, sCD36 did not predict the occurrence of CV events, although in this case the independently associated factors were also older age, diabetes, active smoking, and dialysis therapy (Supplementary Table 5). The results of the model for sCD36 including eGFR are presented in Supplementary Table 6.

**Table 2:** Cardiovascular risk prediction cox regression model for CD5L.

**Predictors**	**HR, 95% CI**	***p*-value**
Diabetes	1.74, 1.1–2.73	**0.017**
CD5L	1.17, 1.0–1.36	**0.045**
Age	1.32, 1.01–1.74	**0.045**
Gender, female	0.86, 0.54–1.36	0.512
Active smoker	2.06, 1.27–3.34	**0.003**
Dyslipidemia	1.04, 0.64–1.69	0.887
Waist circumference	1.17, 0.94–1.45	0.167
Systolic blood pressure	1.13, 0.92–1.39	0.257
CKD 4–5^*^	1.33, 0.82–2.14	0.245
Dialysis^*^	2.18, 1.20–3.95	**0.010**

^*^CKD stage 3 was the reference to assess dialysis and CKD stage 4–5. Abbreviations: CI: confidence interval; CKD: chronic kidney disease; HR: hazard ratio.

### Factors associated with all-cause mortality

Cox proportional hazards models showed that CD5L was an independent predictor of all-cause mortality (HR, 1.22; 95% CI, 1.01–1.48) after adjusting for CV risk factors such as age, sex, diabetes, waist circumference, smoking, systolic blood pressure, dyslipidemia, and CKD stage (Table 3). Other significant independent predictors of death were older age, current smoking, diabetes and dialysis stage (Table 3). The results of the model for CD5L including eGFR are shown in Supplementary Table 7. In contrast, sCD36 did not predict all-cause mortality (Supplementary Table 8), but older age, current smoking, waist circumference, CKD stage 4–5 and dialysis therapy were independent predictors. The results of the model for sCD36 including eGFR are shown in Supplementary Table 9.

**Table 3:** All-cause mortality cox regression model for CD5L.

	**HR, 95% CI**	***p*-value**
Diabetes	1.59, 0.92–2.75	0.099
CD5L	1.22, 1.01–1.48	**0.043**
Age	2.23, 1.50–3.32	**<0.001**
Gender, female	0.65, 0.36–1.15	0.136
Active smoker	1.99, 1.11–3.56	0.020
Systolic blood pressure	1.09, 0.85–1.40	0.490
Dyslipidemia	0.82, 0.47–1.41	0.467
Waist circumference	1.33, 1.02–1.73	**0.034**
CKD 4–5^*^	1.92, 1.06–3.47	**0.032**
Dialysis^*^	4.12, 2.07–8.22	**<0.001**
CD5L::Diabetes	0.69, 0.45–1.07	0.098

^*^CKD stages 4–5 and dialysis taking CKD stage 3 as reference. Abbreviations: CI: confidence interval; CKD: chronic kidney disease; HR: hazard ratio.

### The goodness of fit over adjusted models

We also evaluated whether the adjusted model including CD5L and the traditional CV risk factors improved the prediction of CV events and mortality compared to the model without CD5L. The absolute log-likelihood value showed that inclusion of CD5L in the model slightly improved the predictive ability compared to the model without CD5L (−614.3 vs. −616.0, *p* = 0.065). On the other hand, the absolute log-likelihood value showed that the model, including the interaction between CD5L and diabetes also improved the all-cause mortality model (−424.7 vs. −426.5, *p* = 0.061).

### Competing risks approach

Of the overall population of 1,516 studied subjects, 46 experienced non-CV deaths and 98 CV events, of which 26 were CV-deaths. Considering all-cause mortality as a competing risk, the CV event rate was 1.55 (1.25–1.88) per 1,000 person-years. When considering a CV event as a competing risk, the all-cause mortality rate was 0.69 (0.5–0.92) per 1,000 person-years.

**Figure 1:**
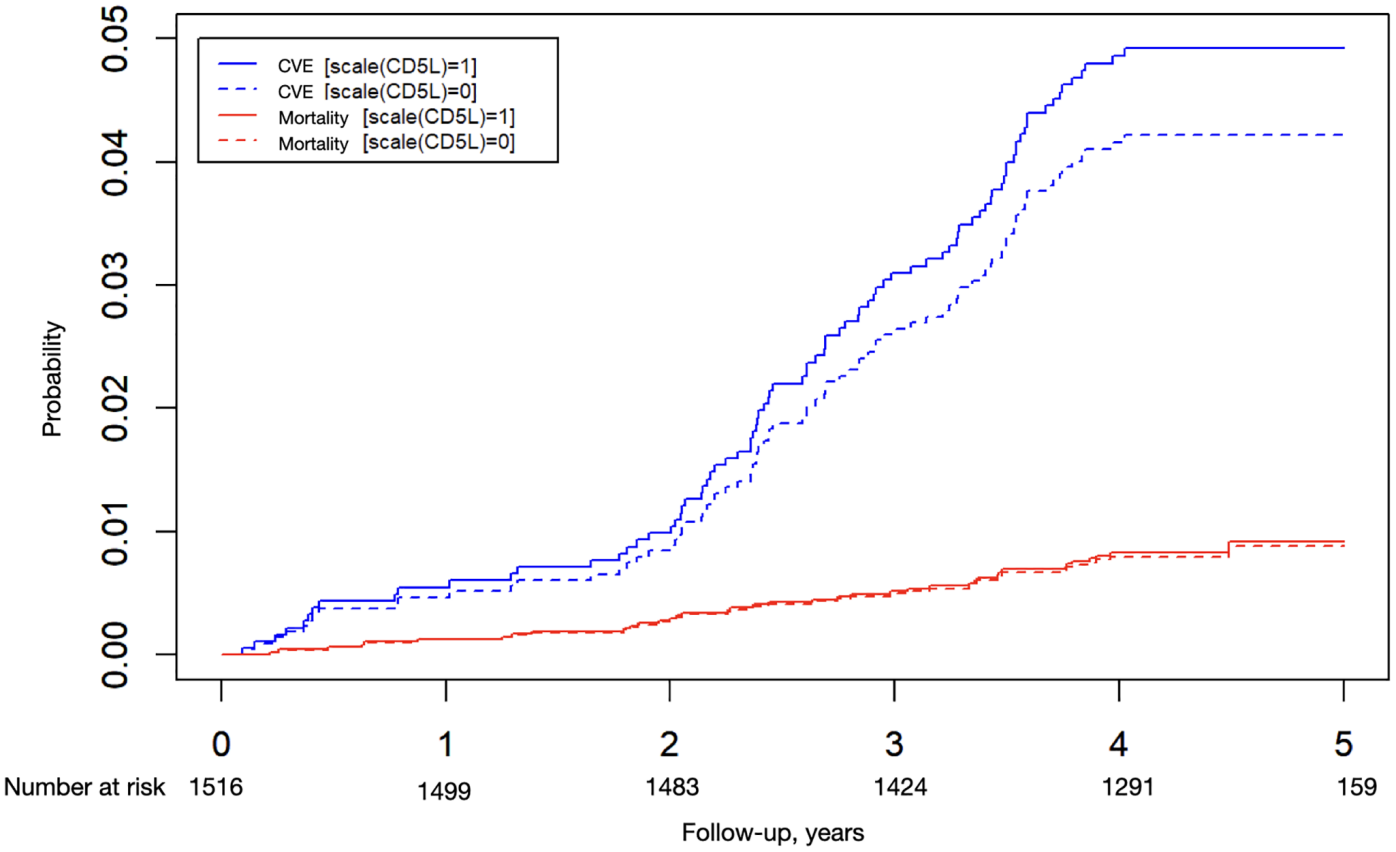
**Probability of a cardiovascular event or all-cause mortality.** Cox models adjusted for competitive risks according to CD5L levels. The continuous line is the median (1) and the dashed line is the median plus one standard deviation (0).

The Cox model revealed that the independent predictive variables for CV events were CD5L (HR, 1.19; 95% CI, 1.01–1.41), diabetes (HR, 1.72; 95% CI, 1.09–2.71), age (HR, 1.29; 95% CI, 0.99–1.68), smoking (HR, 2.03; 95% CI, 1.25–3.28) and dialysis (HR, 2.12; 95% CI, 1.17–3.84). Figure 1 shows that the probability of a CV event was higher with high CD5L concentrations but similar for all-cause mortality according to the Cox model adjusted with competitive risks.

## DISCUSSION

In this multicenter cohort, we found that high circulating CD5L concentrations were associated with CV events and all-cause mortality in individuals with CKD. In contrast, sCD36 was not associated with a risk of CVD or death. This is, to the best of our knowledge, the first time that CD5L has been shown to be associated and could be a potential predictor of CVD and all-cause mortality in individuals with CKD.

### CD5L as a biomarker

Our findings are in line with studies showing that CD5L might be detrimental in metabolic disorders and atherosclerosis, including diabetes and CV events [17, 23]. This could be explained by the fact that CD5L is involved in the pathogenesis of several inflammatory processes as well as in immune homeostasis [23, 24]. Macrophage-derived CD5L enters into the adipocytes via CD36-mediated endocytosis; once inside the cell, it associates with fatty acid synthase (FAS) and catalyzes the synthesis of saturated fatty acids [25]. Saturated fatty acids activate Toll-like receptor (TLR) 4 and induce a response tightly associated with obesity-induced inflammation [26]. Thus, CD5L acts as a key factor in the initiation of obesity-associated chronic inflammation leading to insulin resistance [17, 27], which results in the progression of atherosclerosis and contributes to future CV events [17]. Indeed, CD5L is highly expressed in foam macrophages harvested from atherosclerotic plaques, which supports macrophage survival through inhibition of apoptosis and their consequent accumulation, in turn causing inflammatory responses within the lesion that eventually leads to disease progression [23].

Previous studies in mice have shown that deletion of CD5L reduces the accumulation of pro-inflammatory M1 macrophages in myocardial tissue [28]. Moreover, the depletion of CD5L shows cardiac effects, such as decreased systolic dysfunction, a decreased incidence of cardiac rupture, and reduction of the infarct size during the acute phase after myocardial infarction, in turn resulting in improved survival rates [28, 29]. These findings led to the authors concluding that the inhibition of CD5L could prevent CVD in chronic inflammation and could attenuate the functional impairment after myocardial infarction [28, 29]. In humans, higher CD5L concentrations on epicardial fat secretome were found in male subjects with heart failure who developed atrial fibrillation [30]. Those results also support the hypothesis that CD5L could be useful for predicting CVD. Indeed, recent studies reported that high concentrations of CD5L strongly predict 30-day mortality risk in individuals with bacterial pneumonia [31] and 28-day mortality in adults and pediatric individuals with sepsis [32]. Additionally, a proteomic study found that CD5L is an independent predictor of acute heart rejection, which is a surrogate marker of mortality after transplantation [33]. Our study adds to the literature that CD5L is associated and an independent predictor of mortality in individuals with advanced CKD, i.e., CKD stage 4–5 and dialysis therapy.

### sCD36 as a biomarker

In our study, sCD36 concentrations did not predict CVD or mortality. The usability of sCD36 as a biomarker of CVD is controversial. In one study in children with and without hypercholesterolemia, a high plasma sCD36 concentration was negatively associated with CV risk factors (high body mass index, body weight, waist and hip circumference, systolic blood pressure, and HOMA-IR), suggesting a possible protective effect of sCD36 [22]. Another study also reported possible protective effects of high sCD36 concentrations on metabolic syndrome components in individuals with coronary artery disease [34]. The same study also observed that higher sCD36 concentrations were associated with a lower risk of left ventricular hypertrophy, although it was identified as a potential risk factor of impaired left ventricular diastolic function. In contrast, sCD36 has been reported as an independent risk factor for coronary artery stenosis in elderly individuals with coronary heart disease [35]. Moreover, a study conducted in subjects with moderate to advanced CKD followed for about 5 years found that sCD36 concentrations were an independent predictor of total mortality risk [36]. Finally, a study in individuals with CKD stage 5 also found that higher sCD36 concentrations were associated with increased 3-year mortality, although the association was only significant after correcting for age and gender, but not after additional correction for diabetes and CVD [20].

CD36 represents the primary fatty acid uptake system in the kidneys and appears to play a central role in CKD development and progression [37]. CD36 expression levels are higher in CKD subjects with diabetic nephropathy and kidney damage [38] and are closely associated with CV risk factors such as hyperlipidemia and diabetes [12]. According to previous findings from our group, this role is not reflected by the circulating form of the protein (i.e., sCD36), as we previously reported similar plasma concentrations in individuals with or without diabetes [15] as well as in diabetic and nondiabetic individuals with subclinical carotid atherosclerosis [39].

The discordant results on the association between sCD36 concentrations and atherosclerosis could be explained by several reasons. Among them, the heterogeneity of subjects included in the different studies published so far, ranging from healthy subjects to those with type 2 diabetes, CKD or even with recent CV events; the different definitions of atherosclerosis adopted by researchers, i.e. carotid intima-media thickness (cIMT), subclinical atherosclerotic plaques or plaques associated with a recent CV event; and the different methods used to determine circulating sCD36, especially as there is not a well-characterized or standardized method to evaluate its concentration [34, 40].

The relationship between decreased kidney function and high CV morbidity and mortality has been established in individuals with diabetes [41, 42]. Our finding confirms that individuals with diabetes have a higher susceptibility for CV events than their counterparts without diabetes and, in line with other studies, highlights the relevance of hypertension, smoking, and age in this population [1, 5, 43, 44]. Additionally, and in agreement with previous reports, gender was not identified as a risk factor for CV events or death, although males exhibited slightly higher proportions of both outcomes than females [45, 46]. CD5L was significantly higher in diabetic subjects and those who had a CV event, and tended to be higher in males and those with all-cause mortality.

### Limitations

Our study has several strengths and limitations worth mentioning. The main strength is that it is a large multicenter study from diverse geographic regions in Spain. Moreover, the participants had a variety of kidney disease etiologies, making our results generalizable to the CKD population at large. And no changes were reported in the monitoring protocols of the subjects during the follow-up period. Additionally, all biomarker measurements were performed in the same laboratory to ensure consistency across the whole cohort. Finally, we included a set of well-recognized and well-defined variables to correct for confounding bias. One limitation of the study is that we did not measure tissue-specific biomarker levels. It is therefore possible that elevations in local biomarker levels in the heart or kidneys could have been clinically significant, but not detected from the plasma in our study. The second limitation is that we do not have the urine albumin to creatinine ratio in all patients, a well-known CV/mortality risk factor in CKD. Moreover, the small number of CV deaths that occurred during the follow-up was a significant limitation when analyzing the CV mortality as a single outcome.

## CONCLUSIONS

The identification of specific biomarkers for CVD is crucial for the development of improved diagnostics and personalized treatment strategies. In individuals with CKD, circulating CD5L could improve CVD prediction and may help to identify those at higher CV risk. However, further population studies on CD5L in relation to traditional risk factors are needed to validate its usability as a true CVD biomarker, its predictive validity, and to explore whether its utility is restricted to individuals with CKD.

## METHODS

### Design and study population

This study assessed the predictive ability of CD5L and sCD36 for CV events and mortality in individuals with CKD from the National Observatory of Atherosclerosis in Nephrology (NEFRONA) study [47]. To evaluate the predictive ability of CD5L and sCD36, we included 1,516 participants enrolled in the NEFRONA cohort. The NEFRONA study is a multicenter, prospective observational study. The design, objective, and methods of the NEFRONA study have been described in detail in a previous publication [47]. Briefly, the NEFRONA study included 2,445 subjects between 18 and 75 years of age, with CKD but without prior CVD recruited from 81 hospitals and dialysis clinics throughout Spain between October 2009 and June 2011 [48]. Exclusion criteria were pregnancy, life expectancy below 12 months, any active infection, previous organ transplantation, or known CVD or carotid artery procedure. During a four-year follow-up period, all CV events, CV and non-CV deaths, and kidney transplantations were registered. The study protocol was conducted following the Declaration of Helsinki and approved by the Ethics Committee of Germans Trias i Pujol Hospital and Arnau de Vilanova Hospital. An informed consent form was signed by all the study participants.

### Clinical and biochemical data

Detailed information was collected at baseline including the participant’s medical history, CV risk factors, and medication. The physical examination included standard vital tests and anthropometric measures, such as height, body weight, and waist-hip ratio. Dyslipidemia was defined as a recorded clinical diagnosis or the current use of lipid-lowering medication [47]. Biochemical parameters were obtained from a routine fasting blood test, and the glomerular filtration rate (eGFR) was estimated using the Modification of Diet in Renal Disease Study formula (MDRD-4) [49]. The criteria for diabetes were: a previous diagnosis of diabetes recorded in the individual's medical history, a fasting plasma glucose ≥126 mg/dl or glycated hemoglobin (HbA1c) ≥6.5% (48 mmol/mol) determined by laboratory testing, or a current prescription of any anti-diabetic drug [50].

### Determination of CD5L and sCD36

Plasma concentrations of human CD5L and sCD36 were measured using commercially available kits: CircuLex human AIM/CD5L/Sp ELISA (Medical and Biological Laboratories, Nagova, Aichi-ken, Japan) and sCD36 ELISA (Nordic BioSite, Täby, Sweden); the detection limits were 0.754 ng/mL and 1.95 ng/mL, respectively. Experiments were done in duplicate, with appropriate dilutions according to the manufacturer's instructions. Briefly, samples were incubated in microtiter wells coated with antibodies for either protein for 2 hours. After incubation and washing, a biotinylated antibody conjugated with streptavidin peroxidase was added to the wells for 1 hour. After a second incubation and washing step, the substrate tetramethylbenzidine was added to the wells at room temperature for 20–30 minutes, followed by the addition of sulfuric acid to stop the enzymatic reaction. The absorbance was read at 450 nm using a SpectraMax 340PC384 microplate reader (Molecular Devices, LLC Sunnyvale, USA). The protein concentration was estimated using a four-parameter logistic curve and log-log curve fit, respectively, based on the standards’ measurements.

### Cardiovascular events

Participants were followed-up for 4 years, and data on fatal and non-fatal CV events, death due to any cause, and kidney transplants were recorded by the referring physician [45]. The following CV events were considered, as defined by the International Classification of Diseases, Ninth Revision, Clinical Modification (ICD9-CM): unstable angina, myocardial infarction, transient ischemic attack, cerebrovascular accident, congestive heart failure, arrhythmia, peripheral arterial disease (PAD) or amputation due to PAD, and aortic aneurysm [23]. Cardiovascular mortality causes included myocardial infarction, arrhythmia, congestive heart failure, stroke, abdominal aortic aneurysm, mesenteric infarction, and sudden death. In addition, non-CV mortality from any other causes was recorded; these included deaths caused by neoplasia, accident, infection, non-determined cause, or unknown death.

### Statistical analysis

Categorical variables are presented as frequencies and percentages, while the numerical ones are expressed as the mean with standard deviation or the median with the first and last quartiles (the interquartile range [IQR]).

We used Cox regression models to estimate raw and adjusted hazard ratios (HRs) of CV events in relation to CD5L and sCD36. Potential confounders considered for adjustment in the multivariable models were: diabetes, age, sex, smoking, body mass index, abdominal circumference, systolic blood pressure, dyslipidemia, CKD stage and eGFR. Fine and Gray modeling was used to estimate risk prediction whilst illustrating the effect of competing risk with no CV death and kidney transplantation. Cause-specific HRs were reported from the Cox model, as were subhazard ratio and cumulative incidence function from the Fine and Gray regressions. We tested the proportional hazard assumption graphically and analytically with the test of proportional-hazards assumption. We used a likelihood ratio test to assess if the model with CD5L improved the goodness of fit over CV events and death. Confidence intervals at the 95% level were calculated whenever possible. All analyses were conducted with the free software environment for statistical computing R version 3.5.3 (2019-03-11) for Windows.

## Supplementary Materials

Supplementary Figures

Supplementary Tables
