# Supplementary material for: Circulating CD5L is associated with cardiovascular events and all-cause mortality in individuals with chronic kidney disease
Source: Aging (Albany NY). 2021 Oct 10;13(19):22690–709. doi: 10.18632/aging.203615 (PMC8544330; doi:10.18632/aging.203615)
Supplement: Supplementary Figures [file aging-13-203615-s001.pdf]

SUPPLEMENTARY FIGURES

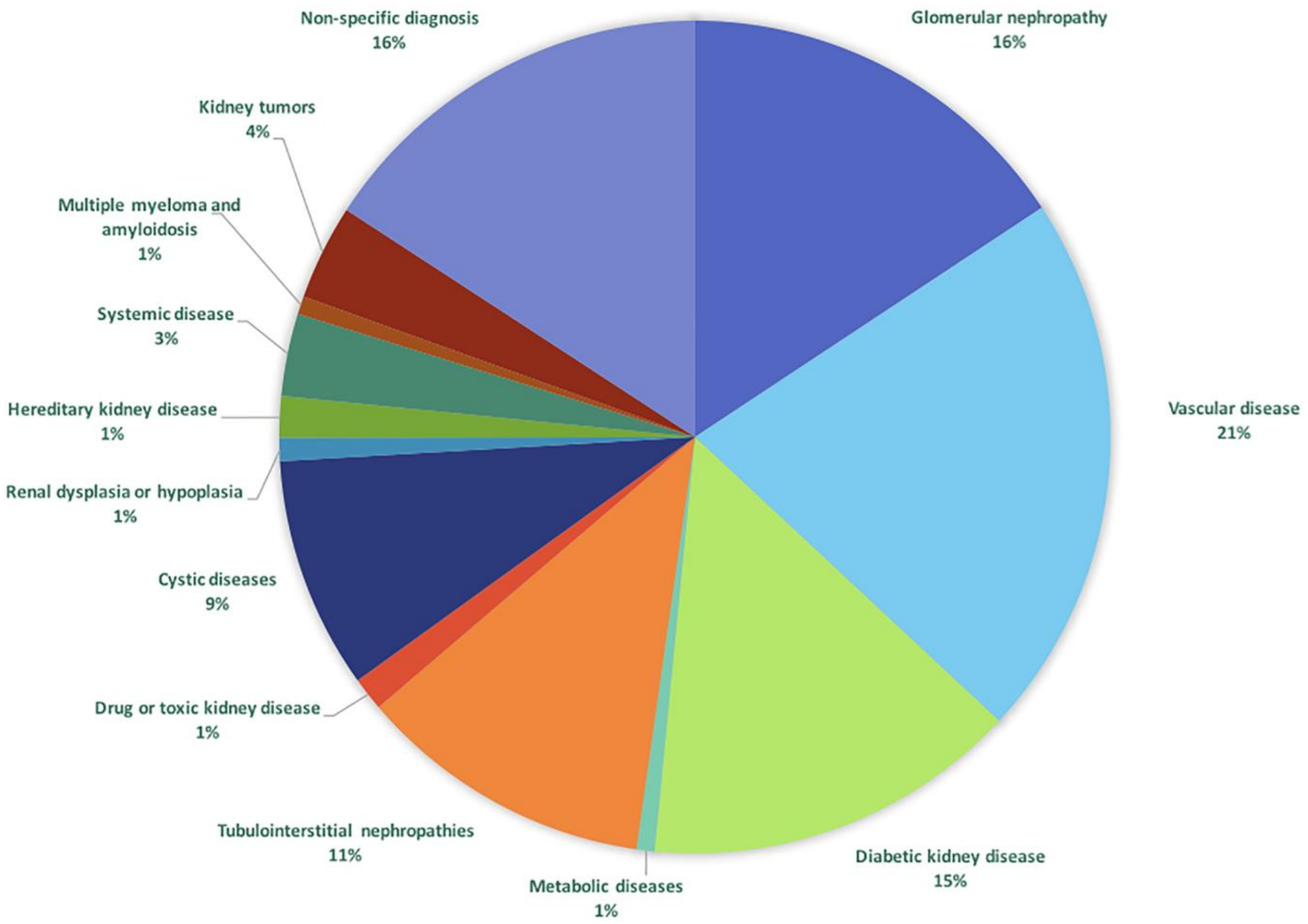

Supplementary Figure 1. Etiology of chronic kidney disease in the study group.

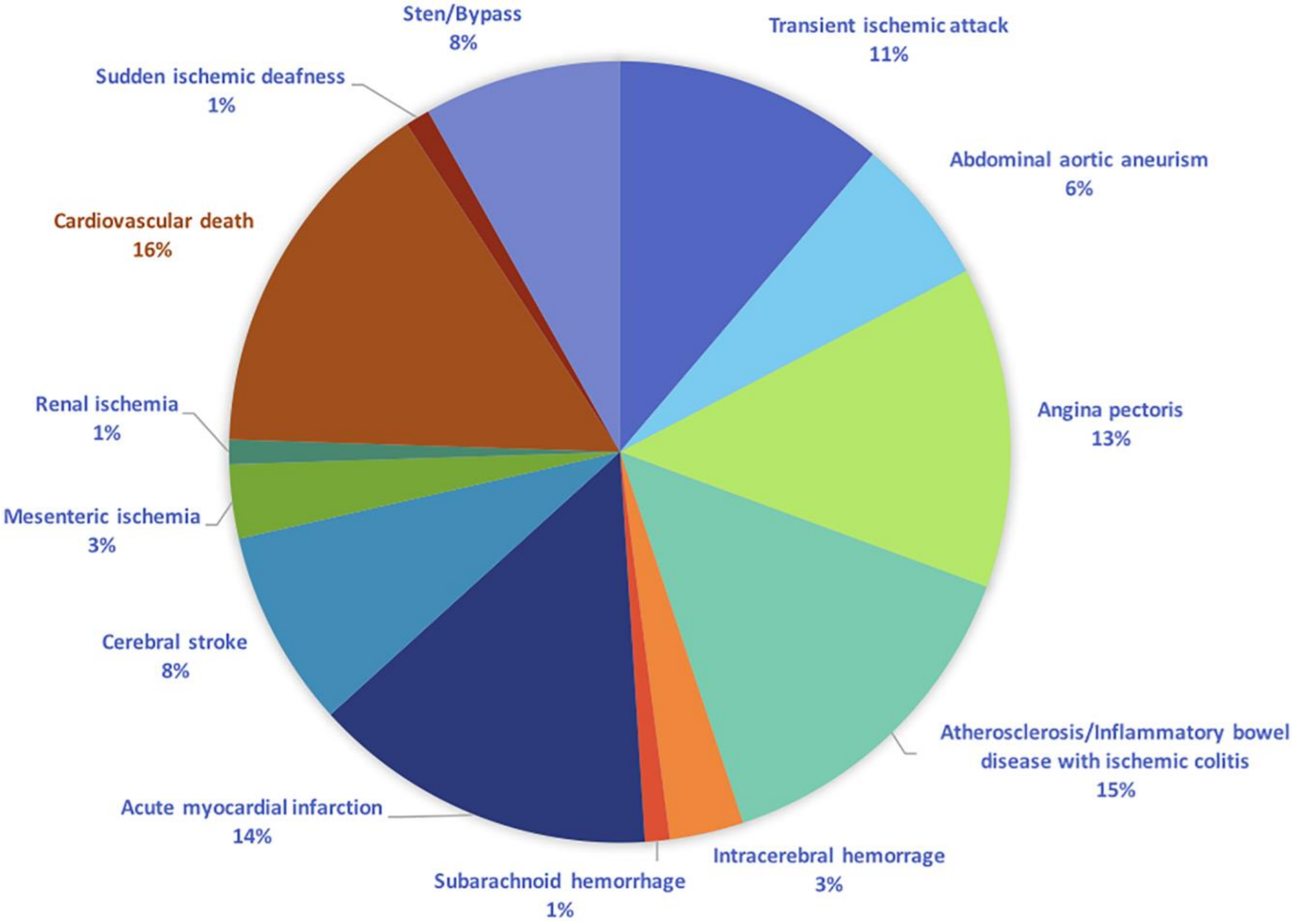

Supplementary Figure 2. Type of cardiovascular events in the study group.
