# Supplementary material for: Circulating CD5L is associated with cardiovascular events and all-cause mortality in individuals with chronic kidney disease
Source: Aging (Albany NY). 2021 Oct 10;13(19):22690–709. doi: 10.18632/aging.203615 (PMC8544330; doi:10.18632/aging.203615)
Supplement: Supplementary Tables [file aging-13-203615-s002.pdf]

SUPPLEMENTARY TABLES

Supplementary Table 1. Clinical characteristics of the study group by gender.

	Male	Female	*p.* overall
N, (%)	926 (61.08)	590 (38.92)	
Diabetes, *n* (%)	265 (28.6)	141 (23.9)	0.050
Body mass index, kg/m^2, median [IQR]	28.1 [25.3; 31.2]	27.8 [24.3; 32.5]	0.975
Waist circumference, cm, median [IQR]	100 [93.0; 108]	95.0 [86.0; 105]	**<0.001**
Active smoker, *n* (%)	218 (23.5)	74 (12.5)	**<0.001**
Hypertension, *n* (%)	854 (92.2)	538 (91.2)	0.533
Dyslipidemia, *n* (%)	659 (71.2)	394 (66.8)	0.080
SBP, mmHg, median [IQR]	142 [130; 157]	139 [126; 156]	**0.004**
DBP, mmHg, median [IQR]	82.0 [75.0; 89.0]	80.0 [73.0; 88.0]	0.095
Age, years, median [IQR]	62.0 [53.0; 69.0]	61.0 [50.0; 68.0]	0.062
Creatinine, mg/dL, median [IQR]	2.11 [1.60; 3.00]	1.90 [1.40; 2.60]	**<0.001**
eGFR, mL/min/1.73 m^2, median [IQR]	34.3 [22.9; 46.9]	29.6 [20.2; 41.3]	**<0.001**
CKD stage, *n* (%)			**<0.001**
CKD-3	447 (48.3)	222 (37.6)	
CKD4-5	306 (33.0)	233 (39.5)	
Dialysis	173 (18.7)	135 (22.9)	
Albumin/creatinine ratio, mg/g, median [IQR]	135 [14.2; 509]	72.8 [10.3; 372]	**0.039**
Total cholesterol, mg/dL, median [IQR]	171 [148; 197]	188 [165; 214]	**<0.001**
HDL cholesterol, mg/dL, median [IQR]	44.0 [36.0; 52.0]	53.0 [45.0; 66.0]	**<0.001**
LDL cholesterol, mg/dL, median [IQR]	98.0 [76.0; 118]	106 [83.5; 126]	**<0.001**
Triglycerides, mg/dL, median [IQR]	124 [94.2; 179]	121 [89.0; 167]	**0.018**
Glucose, mg/dL, median [IQR]	101 [90.0; 116]	94.0 [85.0; 109]	**<0.001**
HbA1c, %, median [IQR]	5.90 [5.38; 6.60]	5.90 [5.30; 7.10]	0.545
Hematocrit, median [IQR]	39.9 [36.0; 43.5]	37.0 [34.7; 39.9]	**<0.001**
Hemoglobin, g/dL, median [IQR]	13.2 [12.0; 14.6]	12.2 [11.4; 13.2]	**<0.001**
CD5L, ng/mL, median [IQR]	2295 [1818; 2875]	2230 [1798; 2888]	0.397
sCD36, ng/mL, median [IQR]	0.85 [0.05; 5.37]	1.20 [0.05; 8.86]	0.028
Cardiovascular event, *n* (%)	65 (7.02)	33 (5.59)	0.320
Kidney transplant, *n* (%)	138 (14.9)	102 (17.3)	0.243
Death			0.093
Alive at the end of follow-up, *n* (%)	874 (94.4)	570 (96.6)	
CV death, *n* (%)	17 (1.84)	9 (1.53)	
Non-CV death, *n* (%)	35 (3.78)	11 (1.86)	

Abbreviations: CDK: chronic kidney disease; CV: cardiovascular; DBP: diastolic blood pressure; eGFR: estimated glomerular filtration rate; HbA1c: glycated hemoglobin; HDL: high-density lipoprotein; IQR: interquartile range; LDL: low-density lipoprotein; SBP: systolic blood pressure.

Supplementary Table 2. Clinical characteristics of the study group by the presence of diabetes.

	No diabetes	Diabetes	*p.* overall
N, (%)	1110 (73.22%)	406 (26.78%)	
Gender, female, *n* (%)	449 (40.5%)	141 (34.7%)	0.050
Body mass index, kg/m^2, median [IQR]	27.5 [24.5; 30.9]	30.0 [26.5; 33.7]	**<0.001**
Waist circumference, cm, median [IQR]	96.0 [89.0; 105]	103 [94.0; 111]	**<0.001**
Active smoker, *n* (%)	216 (19.5%)	76 (18.7%)	0.803
Hypertension, *n* (%)	993 (89.5%)	399 (98.3%)	**<0.001**
Dyslipidemia, *n* (%)	719 (64.8%)	334 (82.3%)	**<0.001**
SBP, mmHg, median [IQR]	139 [127; 154]	146 [132; 162]	**<0.001**
DBP, mmHg, median [IQR]	82.0 [75.0; 89.0]	79.5 [72.0; 86.0]	**<0.001**
Age, years, median [IQR]	61.0 [50.0; 67.0]	65.0 [58.0; 70.0]	**<0.001**
Creatinine, mg/dL, median [IQR]	2.08 [1.51; 2.90]	1.95 [1.54; 2.77]	0.337
eGFR, mL/min/1.73 m^2, median [IQR]	31.6 [21.4; 45.3]	34.3 [22.5; 45.7]	0.306
CKD stage, *n* (%)			**<0.001**
CKD-3	468 (42.2%)	201 (49.5%)	
CKD4-5	381 (34.3%)	158 (38.9%)	
Dialysis	261 (23.5%)	47 (11.6%)	
Albumin/creatinine ratio, mg/g, median [IQR]	83.5 [10.3; 381]	178 [25.4; 687]	**0.001**
Total cholesterol, mg/dL, median [IQR]	180 [156; 208]	171 [144; 197]	**<0.001**
HDL cholesterol, mg/dL, median [IQR]	48.0 [40.0; 59.0]	44.0 [36.0; 54.0]	**<0.001**
LDL cholesterol, mg/dL, median [IQR]	105 [83.9; 125]	90.0 [71.6; 111]	**<0.001**
Triglycerides, mg/dL, median [IQR]	118 [89.8; 164]	140 [101; 205]	**<0.001**
Glucose, mg/dL, median [IQR]	94.0 [86.0; 103]	134 [108; 169]	**<0.001**
HbA1c, %, median [IQR]	5.50 [5.10; 5.80]	6.90 [6.20; 8.00]	**<0.001**
Hematocrit, median [IQR]	38.8 [35.5; 42.3]	37.7 [34.8; 41.1]	**0.002**
Hemoglobin, g/dL, median [IQR]	12.8 [11.8; 14.2]	12.5 [11.5; 13.8]	**0.005**
CD5L, ng/mL, median [IQR]	2206 [1782; 2802]	2421 [1899; 3105]	**<0.001**
sCD36, ng/mL, median [IQR]	0.83 [0.05; 5.93]	1.31 [0.05; 7.57]	0.311
Cardiovascular event, *n* (%)	55 (4.95%)	43 (10.6%)	**<0.001**
Kidney transplant, *n* (%)	200 (18.0%)	40 (9.85%)	**<0.001**
Death			**0.013**
Alive at the end of follow-up, *n* (%)	1068 (96.2)	376 (92.6%)	
CV death, *n* (%)	16 (1.44%)	10 (2.46%)	
Non-CV death, *n* (%)	26 (2.34%)	20 (4.93%)	

Abbreviations: CDK: chronic kidney disease; CV: cardiovascular; DBP: diastolic blood pressure; eGFR: estimated glomerular filtration rate; HbA1c: glycated hemoglobin; HDL: high-density lipoprotein; IQR: interquartile range; LDL: low-density lipoprotein; SBP: systolic blood pressure.

Supplementary Table 3. Clinical characteristics of the study group by the presence of a cardiovascular event during follow-up.

	NoCVE	CVE	*p.* overall
N, (%)	1418 (93.54%)	98 (6.46%)	
Diabetes, *n* (%)	363 (25.6%)	43 (43.9%)	**<0.001**
Gender, female, *n* (%)	557 (39.3%)	33 (33.7%)	0.320
Body mass index, kg/m^2, median [IQR]	28.0 [24.9; 31.7]	29.3 [25.7; 32.4]	0.117
Waist circumference, cm, median [IQR]	98.0 [90.0; 107]	102 [93.0; 110]	**0.009**
Active smoker, *n* (%)	263 (18.5%)	29 (29.6%)	**0.011**
Hypertension, *n* (%)	1298 (91.5%)	94 (95.9%)	0.180
Dyslipidemia, *n* (%)	980 (69.1%)	73 (74.5%)	0.315
SBP, mmHg, median [IQR]	140 [128; 156]	146 [131; 163]	0.125
DBP, mmHg, median [IQR]	81.0 [74.0; 89.0]	83.0 [72.0; 89.8]	0.844
Age, years, median [IQR]	62.0 [51.0; 68.0]	64.0 [54.0; 69.8]	0.074
Creatinine, mg/dL, median [IQR]	2.00 [1.51; 2.87]	2.13 [1.70; 3.14]	0.093
eGFR, mL/min/1.73 m^2, median [IQR]	32.5 [21.9; 45.7]	30.1 [19.6; 39.1]	0.089
CKD stage, *n* (%)			**0.046**
CKD-3	634 (44.7%)	35 (35.7%)	
CKD4-5	505 (35.6%)	34 (34.7%)	
Dialysis	279 (19.7%)	29 (29.6%)	
Albumin/creatinine ratio, mg/g, median [IQR]	96.0 [11.9; 448]	212 [91.1; 1000]	**0.003**
Total cholesterol, mg/dL, median [IQR]	178 [154; 205]	169 [139; 206]	0.185
HDL cholesterol, mg/dL, median [IQR]	47.0 [39.0; 58.5]	43.0 [33.5; 51.2]	**<0.001**
LDL cholesterol, mg/dL, median [IQR]	101 [80.4; 122]	93.5 [67.3; 117]	0.054
Triglycerides, mg/dL, median [IQR]	122 [91.0; 174]	135 [104; 184]	0.088
Glucose, mg/dL, median [IQR]	98.0 [87.0; 112]	103 [91.0; 152]	**0.002**
HbA1c, %, median [IQR]	5.80 [5.30; 6.70]	6.80 [6.00; 8.00]	**<0.001**
Hematocrit, median [IQR]	38.6 [35.3; 42.0]	37.8 [34.0; 41.8]	0.090
Hemoglobin, g/dL, median [IQR]	12.8 [11.8; 14.1]	12.4 [11.3; 14.0]	0.066
CD5L, ng/mL, median [IQR]	2245 [1806; 2847]	2571 [1910; 3314]	**0.004**
sCD36, ng/mL, median [IQR]	0.96 [0.05; 6.79]	1.31 [0.05; 6.66]	0.347
Kidney transplant, *n* (%)	229 (16.1%)	11 (11.2%)	0.251
Death			**<0.001**
Alive at the end of follow-up, *n* (%)	1374 (96.9%)	70 (71.4%)	
CV death, *n* (%)	0 (0.00%)	26 (26.5%)	
Non-CV death, *n* (%)	44 (3.10%)	2 (2.04%)	

Abbreviations: CDK: chronic kidney disease; CV: cardiovascular; DBP: diastolic blood pressure; eGFR: estimated glomerular filtration rate; HbA1c: glycated hemoglobin; HDL: high-density lipoprotein; IQR: interquartile range; LDL: low-density lipoprotein; SBP: systolic blood pressure.

Supplementary Table 4. Cardiovascular risk prediction cox regression model for CD5L.

Predictors	HR, 95% CI	*p-value*
Diabetes	1.56, 0.94– 2.59	0.083
CD5L	1.23, 1.02–1.48	**0.030**
Age	1.26, 0.91–1.73	0.161
Gender, female	0.86, 0.51–1.46	0.570
Active smoker	2.50, 1.47–4.26	**0.001**
Dyslipidemia	1.64, 0.85–3.16	0.138
Waist circumference	1.24, 0.97–1.58	0.091
Systolic blood pressure	1.24, 0.98–1.58	0.073
eGFR (MDRD4)	0.76, 0.59–0.98	**0.037**

Abbreviations: CI: confidence interval; eGFR: estimated glomerular filtration rate; HR: hazard ratio; MDRD: Modification of Diet in Renal Disease.

Supplementary Table 5. Cardiovascular risk prediction cox regression model for sCD36.

Predictors	HR, 95% CI	*p*
Diabetes	1.83 1.17–2.87	**0.009**
sCD36	1.03 0.83–1.29	0.772
Age	1.36 1.04–1.79	**0.025**
Gender, female	0.88 0.55–1.39	0.574
Smoker	2.08 1.28–3.38	**0.003**
Dyslipidemia	1.03 0.63–1.68	0.912
Waist circumference	1.17 0.94–1.46	0.148
Systolic blood pressure	1.12 0.91–1.39	0.272
CKD4–5*	1.37 0.85–2.20	0.198
Dialysis*	2.45 1.37–4.38	**0.003**

*CKD stages 4–5 and dialysis taking CKD stage 3 as reference. Abbreviations: CI: confidence interval; HR: hazard ratio; CKD: chronic kidney disease.

Supplementary Table 6. Cardiovascular risk prediction cox regression model for sCD36.

Predictors	HR, 95% CI	*p*
Diabetes	1.69, 1.03–2.77	**0.039**
sCD36	1.07, 0.83–1.38	0.586
Age	1.29, 0.94–1.78	0.113
Gender, female	0.89, 0.53–1.51	0.5671
Smoker	2.54, 1.49–4.35	**0.001**
Dyslipidemia	1.63, 0.85–3.13	0.145

Waist circumference	1.26, 0.98–1.61	0.070
Systolic blood pressure	1.25, 0.98–1.59	0.069
eGFR (MDRD4)	0.75, 0.58–0.96	**0.025**

Abbreviations: CI: confidence interval; eGFR: estimated glomerular filtration rate; HR: hazard ratio; MDRD: Modification of Diet in Renal Disease.

Supplementary Table 7. All-cause mortality cox regression model for CD5L.

	HR, 95% CI	*p-value*
Diabetes	1.71, 0.92–3.16	0.088
CD5L	1.25, 0.89–1.75	0.202
CD5L::Diabetes	0.73, 0.43–1.27	0.268
Age	0.45, 0.21–0.95	**0.002**
Gender, female	0.63, 0.36–1.13	**0.037**
Active smoker	2.17, 1.11–4.24	**0.023**
Dyslipidemia	1.38, 0.63– 3.02	0.421
Waist circumference	1.42, 1.03–1.96	**0.031**
Systolic blood pressure	1.11, 0.82–1.50	0.498
eGFR (MDRD4)	0.63, 0.46–0.88	**0.007**

Abbreviations: CKD: chronic kidney disease; CI: confidence interval; eGFR: estimated glomerular filtration rate; HR: hazard ratio; MDRD: Modification of Diet in Renal Disease. *CKD stage 3 was the reference to assess dialysis and CKD stage 4–5.

Supplementary Table 8. All-cause mortality cox regression model for CD36.

**Predictors**	**HR 95% CI**	***p***
Diabetes	1.50 0.87–2.58	0.143
CD36	1.12 0.89–1.41	0.320
Age	2.28 1.53–3.39	**<0.001**
Gender, female	0.62 0.35–1.10	0.103
Smoker	2.07 1.15–3.72	**0.015**
Systolic blood pressure	1.08 0.84–1.38	0.562
Dyslipidemia	0.83 0.48–1.45	0.517
Waist circumference	1.31 1.01–1.70	**0.046**
CKD 4–5*	1.96 1.09–3.55	**0.026**
Dialysis*	4.40 2.26–8.58	**<0.001**

Abbreviations: CKD: chronic kidney disease; confidence interval; HR: hazard ratio. *CKD stages 4–5 and dialysis taking CKD stage 3 as reference.

Supplementary Table 9. All-cause mortality cox regression model for CD36.

Predictors	HR 95% CI	*p*
Diabetes	1.70, 0.92–3.14	0.091
CD36	1.18, 0.89–1.55	0.245
Age	2.27, 1.38–3.74	**0.001**
Gender, female	0.43, 0.20–0.91	**0.027**
Smoker	2.21, 1.13–4.32	**0.021**
Dyslipidemia	1.35, 0.62–2.95	0.447
Waist circumference	1.41, 1.02–1.94	**0.036**
Systolic blood pressure	1.09, 0.81–1.47	0.578
eGFR (MDRD4)	0.63, 0.46–0.87	**0.006**

Abbreviations: CI: confidence interval; eGFR: estimated glomerular filtration rate; HR: hazard ratio; MDRD: Modification of Diet in Renal Disease. *CKD stages 4–5 and dialysis taking CKD stage 3 as reference.
